# Enhancing dentin bonding quality through Acetone wet-bonding technique: a promising approach

**DOI:** 10.3389/fbioe.2023.1309503

**Published:** 2023-12-07

**Authors:** Shikai Zhao, Zhiyi Zhu, Jian Yu, Chenmin Yao, Miaoyang Yu, Hongye Yang, Cui Huang

**Affiliations:** ^1^ State Key Laboratory of Oral & Maxillofacial Reconstruction and Regeneration, Key Laboratory of Oral Biomedicine Ministry of Education, Hubei Key Laboratory of Stomatology, School and Hospital of Stomatology, Wuhan University, Wuhan, China; ^2^ Department of Stomatology, Maternal and Child Health Hospital of Hubei Province, Tongji Medical College, Huazhong University of Science and Technology, Wuhan, China

**Keywords:** dentin, bonding, adhesive, interface, Acetone

## Abstract

**Objective:** This paper aimed to assess the impact of the acetone wet-bonding (AWB) technique on dentin bonding and to investigate its potential underlying mechanisms.

**Materials and Methods:** Caries-free third molars were sliced, ground, etched, water-rinsed. Then the specimens were randomly allocated to four groups according to the following pretreatments: 1. water wet-bonding (WWB); 2. ethanol wet-bonding (EWB); 3. 50% (v/v) acetone aqueous solution (50%AWB); 4. 100% acetone solution (AWB). Singlebond universal adhesive was then applied and composite buildups were constructed. The microtensile bond strength (MTBS), failure modes and interface nanoleakage were respectively evaluated after 24 h of water storage, 10,000 times of thermocycling or 1-month collagenase ageing. *In situ* zymography and contact angle were also investigated.

**Results:** Acetone pretreatment preserved MTBS after thermocycling or collagenase ageing (*p <* 0.05) without affecting the immediate MTBS (*p >* 0.05). Furthermore, AWB group manifested fewer nanoleakage than WWB group. More importantly, the contact angle of the dentin surfaces decreased significantly and collagenolytic activities within the hybrid layer were suppressed in AWB group.

**Conclusion:** This study suggested that the AWB technique was effective in enhancing the dentin bond durability by increasing the wettability of dentin surface to dental adhesives, removing residual water in the hybrid layer, improving the penetration of adhesive monomer, and inhibiting the collagenolytic activities.

**Clinical significance:** The lifespan of adhesive restorations would be increased by utilization of acetone wet-bonding technique.

## 1 Introduction

Since the 20th century, adhesive techniques played a dominant role in aesthetic dentistry (Drummond, 2008). Although the manufacturers claim that dentin adhesive system has evolved as far as eighth generation (Taneja et al., 2017), the dentin bonding durability remains inadequate despite its immediate effectiveness (Deligeorgi et al., 2001). Consequently, almost half of all aesthetic restorations require repair over a span of a decade and dentists find themselves dedicating a significant portion, approximately 60%, of their work hours to replace them (Deligeorgi et al., 2001). Therefore, urgent measures must be taken to enhance the durability of dentin bonding to prolong the service life of adhesive restorations.

The degradation of the hybrid layer at adhesive-dentin bonding interface is widely regarded as the chief contributing factor for the decline of bond durability (Tjaderhane et al., 2013). Adhesive hydrolysis, host-derived enzymatic degradation of collagen, inadequate infiltration of adhesive monomers, and secondary caries are possible threats that could lead to hybrid layer degradation (Breschi et al., 2018; Stewart and Finer, 2019). Various of measures, such as ethanol wet-bonding, biomimetic remineralization, collagen cross-linkers, and MMP inhibitors, have been suggested as potential options to protect the integrity of bonding interfaces and achieve reliable bonding durability (Ekambaram et al., 2014; R; Guo et al., 2021; Yang et al., 2016; Yi et al., 2019; Yu et al., 2022).

During dentin bonding, the dentin surface is firstly demineralized using phosphoric acid to create exposed collagen matrix with nanometer porosities. To prevent etched collagen from collapsing, water is needed to enter and maintain the interfibrillar spaces. This facilitates the infiltration of the adhesive monomers into the nanosized porosities of the collagen matrix for interlocking (Sartori et al., 2015). Consequently, the “wet bonding technique” was introduced to achieve higher bonding effectiveness, enhanced sealing of dentinal tubules and less post-operative discomfort (D. H. Pashley et al., 2011). But residual water can induce phase separation of the adhesive monomer (Spencer and Wang, 2002), leading to the formation of hydrophilic comonomer in the hybrid layer. This, in turn, results in the exclusion of hydrophobic monomers and hydrolyzation of the collagen (Wang and Spencer, 2003). Additionally, existing water in the collagen matrix actives endogenous enzymes, which triggers the proteolytic degradation process of the unprotected collagen (D. H. Pashley et al., 2004; Sartori et al., 2015). To address these issues, another solvent, ethanol, was introduced to replace water. Satisfactory laboratory results were obtained in *in vitro* studies by utilizing the “ethanol wet bonding technique”, which requires experimental hydrophobic dental adhesive to contain ethanol of incremental concentrations (Ekambaram et al., 2014).

Acetone is a colorless, flammable and volatile liquid with a low-boiling point that evaporates rapidly and has a faint aroma. It is renowned as one of the most commonly employed solvents in various applications because it can readily blend with most organic solvents and completely with water. Moreover, it can be naturally synthesized by the human body during metabolic processes. Therefore, it has been considered a Generally Recognised As Safe (GRAS) substance when found in desserts, beverages and baked goods (Besinis et al., 2016). Acetone is a polar aprotic solvent capable of dissolving both polar and nonpolar compounds. It dissolves most monomers in dental adhesives (Ekambaram, Yiu and Matinlinna, 2015). Owing to its high dipole moment and good evaporation capacity (Abate, Rodriguez and Macchi, 2000), acetone possesses excellent water-removal capacities, earning it the name “water-chaser” (Jacobsen and Soderholm, 1995; Van Landuyt et al., 2007). Due to the fact that ethanol possesses a viscosity three times greater than that of acetone, dental adhesives based on acetone are less viscous than those based on ethanol, which promotes the acetone’s infiltration into the demineralized dental collagen matrix (Faria-e-Silva et al., 2013). Previous studies examining acetone-based adhesives did not exclusively employ acetone as a sole preconditioning agent. Moreover, crucial parameters such as microtensile bond strength after ageing, nanoleakage, zymography and contact angle were not reported, all of which are essential for assessing dentin bond durability and dentin surface wettability (Irmak et al., 2016; X; Li, et al., 2004). Therefore, it is promising to explore the feasibility of “acetone wet-bonding” to improve the dentin bonding stability, especially when contrasted with conventional wet boding technique.

Thus, the objective of this study was to assess the impact of acetone pretreatment on dentin bonding in comparison to water and ethanol and explore possible mechanisms. The null hypotheses posited that acetone pretreatment would not result in (1) an enhancement in bond strength and a decrease in nanoleakage within the hybrid layer, and (2) any impact on collagen hydrolytic degradation within the hybrid layer, as well as the contact angle of the dentin surface.

## 2 Materials and methods

### 2.1 Chemicals and reagents

**FIGURE 1:**
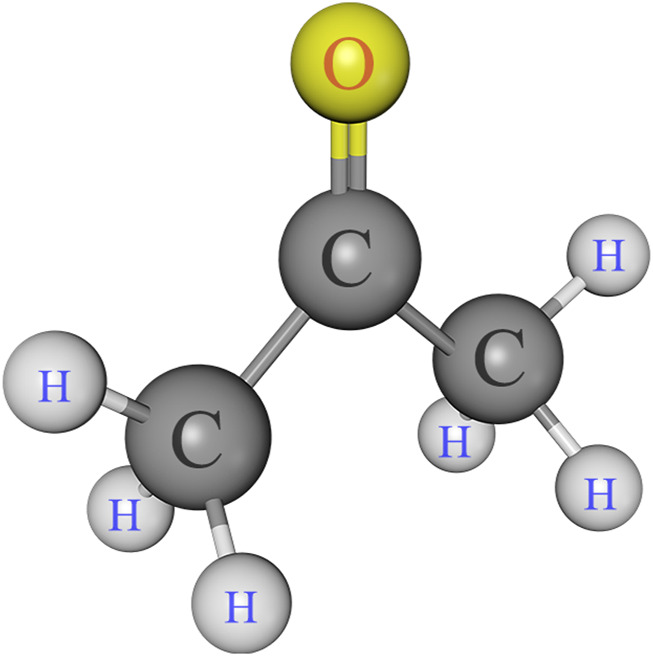
Chemical structure of acetone.

Singlebond universal adhesive and 35% phosphoric acid gel were purchased from 3M ESPE (St. Paul, MN, United States). Charism resin composite was supplied by Heraeus Kulzer (Hanau, Germany). Ethanol and acetone were provided by Aladdin Bio-Chem Technology (Shanghai, China). Kits of gelatinase/collagenase assay (E12055) was supplied by Molecular Probes (Invitrogen, Eugene, OR, United States). The chemicals and reagents were utilized in their as-received condition. Figure 1 displays the chemical structure of acetone.

### 2.2 Specimen preparation and bonding protocols

Sixty sound caries-free third molars were promptly cleansed and subsequently preserved in a 0.1% thymol solution at a temperature of 4 °C before utilization. Approval from the Ethics Committee for Human Studies of the School and Hospital of Stomatology, Wuhan University, China was secured for the utilization of the human third molars. A 50% (v/v) acetone aqueous solution was promptly prepared prior to use by blending acetone in sterilized deionized water.

To reveal the mid-coronal dentin surfaces, the teeth were cut below the enamel-dentinal junction using a diamond saw (Isomet, Buehler Ltd., Lake Bluff, IL, United States). The standardized smear layer was produced by wet-polishing the dentin surfaces with 600-grit SiC paper for 60s. Subsequently, the specimens underwent a 15 s etching with 35% phosphoric acid, followed by thoroughly rinsing with deionized water. The surfaces were then blot-dried and treated with four primer solutions by using a micro-brush for 60s. The specimens were randomly distributed into four groups, with 15 teeth in each group, based on the different solutions as follows.

Group 1: deionized water (water wet-bonding, WWB group).
Group 2: absolute ethanol (ethanol wet-bonding, EWB group).
Group 3: 50% (v/v) acetone aqueous solution (water-acetone wet-bonding, 50%AWB group).
Group 4: absolute acetone (acetone wet-bonding, AWB group).

**FIGURE 2:**
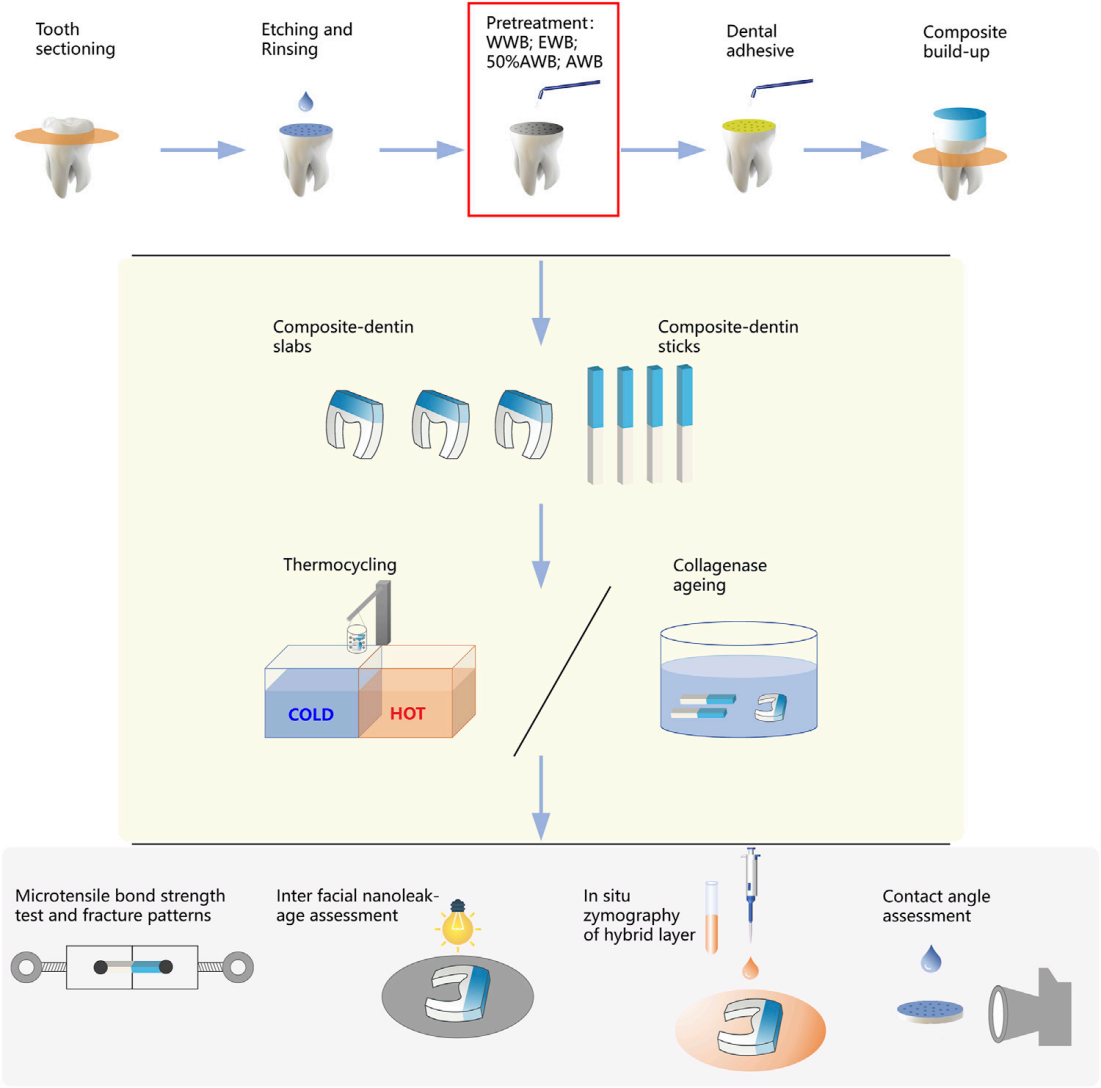
Schematic diagram illustrating the workflow of this study, including sample preparation, measurement of microtensile bond strength, nanoleakage, *in situ* zymography and contact angle.

Following gentle blotting with filter paper, the dentin surface was rubbed for 20 s by a micro-brush dipped with Singlebond Universal (St. Paul, MN, United States). Then, the surfaces were subjected to 5 s of air blowing, and a LED light-curing (Bisco Inc., Schaumburg, IL, United States) was employed to polymerize the adhesive for 20 s. A 5-mm thick layer of composite (Charisma, Kulzer, Germany) was gradually added and polymerized for 20 s at a time. All the experimental procedures were performed by the same highly skilled clinician. Figure 2 illustrates the entire experimental procedure.

### 2.3 Microtensile bond strength test

The bonded teeth were immersed in water for 24 h. Then the specimens were longitudinally sectioned, yielding slabs with a thickness measuring 0.9 mm. To assess nanoleakage, six middle slabs from each group (n = 6) were randomly chosen and stored. Additionally, two middle slabs from each group (n = 2) were randomly chosen and set aside for *in situ* zymography. The remaining slabs were subsequently cut to produce beams measuring 0.9 mm × 0.9 mm. Following careful evaluation, unqualified beams, for example, those with enamel residual or positioned at the periphery were excluded. Twelve eligible beams were then selected from each tooth sample. Out of these, four (total *n* = 36) underwent MTBS immediately, another four (total n = 36) were subjected to 10,000 runs of thermocycling, and the remaining four (total *n* = 36) were assessed after 1 month of collagenase ageing. For the thermocycling procedure, the samples were inserted into a thermocycling apparatus (Thermo Fisher, Newington, United States) and subjected to alternate temperature cycles of 5°C and 55°C every 15 s, totaling 10,000 cycles (Deng et al., 2014). Regarding collagenase ageing, the specimens were submerged in a 0.1 mg/mL collagenase solution at 37°C in a dark environment. The ageing solution was produced by dissolving collagenase from *Clostridium histolyticum* in an artificial saliva medium (Zhang et al., 2020). The ageing solution was replenished periodically, every 3 days.

The chosen beams were individually affixed to a microtensile testing apparatus (Bisco Inc., Schaumburg, IL, United States). They were subjected to tension until fracture occurred, with a cross-head speed set at 1 mm per minute. Comprehensive data were meticulously documented, inclusive of the prematured detached beams. However, it is noteworthy that the values of these samples were excluded from the statistical analysis due to the fact that premature failure constituted less than 3 percent of the total samples tested in each group. Following the determination of the maximum load (N), the dimensions of each beam (mm^2^) were assessed utilizing a digital caliper. Subsequently, the ultimate microtensile bond strength (MPa) was computed accordingly.

### 2.4 Fracture mode analysis

Following the MTBS assessment, the fractured beams were collected and subjected to drying. The surface of each beam underwent sputter-coating with Au-Pd alloy (JFC-1600, JEOL, Tokyo, Japan), and they were then subjected to analysis utilizing field-emission scanning electron microscopy (FESEM, Zeiss sigma, Jena, Germany). The fracture patterns were categorized into four types (Cova et al., 2011): (1) adhesive failure/A; (2) cohesive failure in dentin/CD; (3) cohesive failure in composite/CC; (4) mixed failure/M.

### 2.5 Interfacial nanoleakage evaluation of adhesive-dentin interface

Six middle slabs selected from each group, which had been stored, were randomly assigned for immediate evaluation (*n* = 2), evaluation after thermocycling (*n* = 2), or evaluation after 1 month of collagenase ageing (*n* = 2). The specimens underwent a double layer of nail polish application, with careful attention to maintain a consistent 1 mm distance from the bonded interface. Subsequently, all slabs were immersed in a 50% (w/v) silver nitrate/ammoniacal solution in the absence of light for a duration of 24 h. Following this, they underwent a thorough rinse in deionized water and were then immersed in a photo developing solution, exposed to fluorescent lighting for a duration of 8 h. They were subsequently wet-ground using 600, 800, 1,200, 2000 and 3000-grit silicon carbide papers. All specimens were subjected to ultrasonic cleaning, followed by drying and sputter-coating with carbon (JFC-1600, JEOL, Tokyo, Japan) before the final evaluation.

Using field-emission scanning electron microscopy (FESEM) in the back-scattered electron mode, the interfacial nanoleakage in all slabs was evaluated. In each slab, a total of 10 randomly chosen field-of-views were captured spanning the entire bonding interface and documented (20 images per subgroup). NIH ImageJ software (Bethesda, MD, United States) was employed to calculate the nanoleakage percentage of silver nitrate deposition within the dentin-adhesive layer. The interfacial nanoleakage was individually assessed by two examiners, expressed as a percentage and scored on a scale of 0–4, following a previously reported protocol (Li et al., 2017): 0, indicating no nanoleakage; 1, representing less than 25% nanoleakage; 2, denoting nanoleakage between 25% and 50%; 3, signifying nanoleakage between 50% and 75%; 4, indicating more than 75% nanoleakage. Kappa test was conducted to evaluate the consistency between the two examiners (K = 0.88).

### 2.6 Zymography of the hybrid layer

Two of the middle slabs, which had been preserved, were randomly chosen from each group for *in situ* zymography. The manufacturer’s instructions were followed in the preparation of the gelatinase/collagenase assay kit, which contained fluorescein-tagged DQ gelatin conjugate. After wet-burnishing the specimens to achieve a thickness of approximately 50 μm, they were carefully mounted on a microscope slide. Subsequently, the specimens were covered with coverslips after applying the gelatin mixture onto these slabs. In the next 24 h, the prepared specimens were incubated in a darkened, humid chamber at a temperature of 37°C.

The slabs underwent examination through confocal laser scanning microscopy (CLSM, Leica, Wetzlar, Germany) in fluorescence mode, employing a ×40 objective lens with a numerical aperture of 0.95. The excitation/emission wavelengths were adjusted at 488/530 nm. Characteristic images were randomly captured from each slab, all at the same z layer. This was done to assess the activity of endogenous gelatinolytic proteases, encompassing MMPs and cysteine cathepsins, determined by the level of green fluorescence (Gou et al., 2018).

### 2.7 Surface contact angle measurements

Twelve third molars were used to acquire dentin disks, each 0.5 mm in thickness, by sectioning them below the enamel-dentinal junction with a low-speed diamond saw. These specimens were then subjected to wet-burnishing with 600, 800, 1,200, and 2000-grit SiC papers and 0.25 μm diamond paste, followed by a 5-min ultrasonic cleaning, a 15-s etching with 35% phosphoric acid gel, thorough rinsing with deionized water, and blot drying with filter papers. The disks were subsequently distributed randomly into four groups, each consisting of 6 disks: Group 1 (WWB); Group 2 (EWB); Group 3 (50%AWB); Group 4 (AWB). To measure the contact angle of each specimen, a Contact Angle System OCA (Dataphysics Instruments, Filderstadt, Germany) was utilized. A 5 μL of Singlebond universal adhesive was meticulously applied onto the surface immediately after blot-drying. The droplet’s image was captured with a digital camera, and the contact angle was subsequently measured, keeping the distance between the dentin surface and the tip constant.

## 3 Results

### 3.1 Microtensile bond strength

**FIGURE 3:**
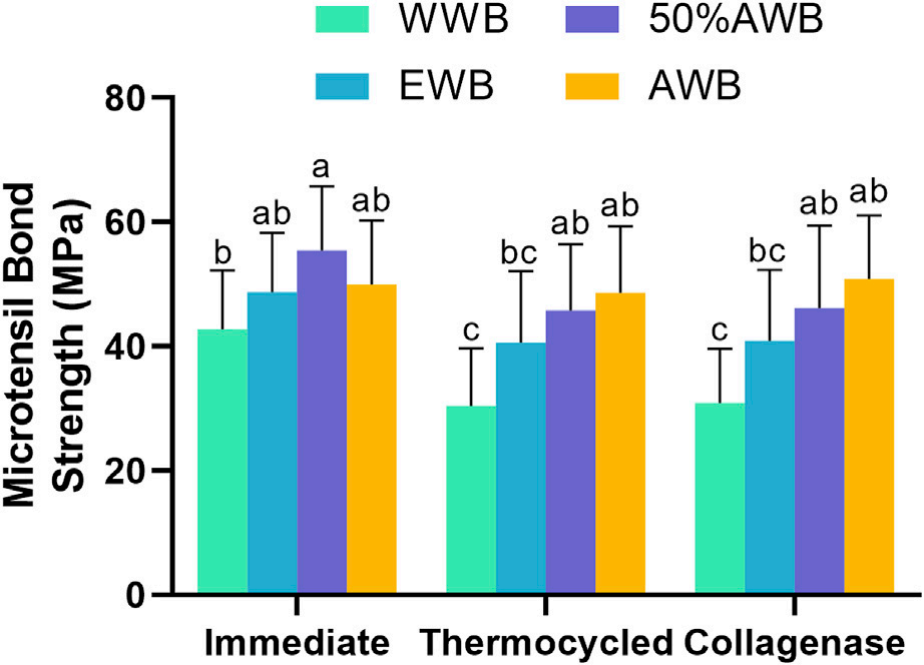
Means and standard deviations of microtensile bond strength (MTBS) for each group (groups with the same superscripts are not statistically significant (*p >* 0.05)).

The mean MTBS values obtained from the four groups are depicted in Figure 3. Two-factor ANOVA indicated that both pretreatments (F = 24.730, *p <* 0.001) and ageing methods (F = 11.408, *p <* 0.001) had a significant impact on dentin bond strength. For immediate bond strength, there was no significant difference observed in the EWB and AWB groups compared with the WWB group (*p >* 0.05). On the contrary, the 50%AWB group exhibited the highest value (*p <* 0.05).

In terms of bond strength after thermocycling or collagenase ageing, the AWB and 50%AWB groups displayed significantly higher values than the WWB group (*p <* 0.05). While there was no significant difference among the experimental groups (*p >* 0.05), it is worth noting that the AWB and 50%AWB groups had higher absolute values than the EWB group. The bond strength of the WWB group witnessed a significant decrease after thermocycling or collagenase ageing. However, the bond strength of the AWB group remained unaltered (*p >* 0.05).

### 3.2 Fracture mode analysis

**FIGURE 4:**
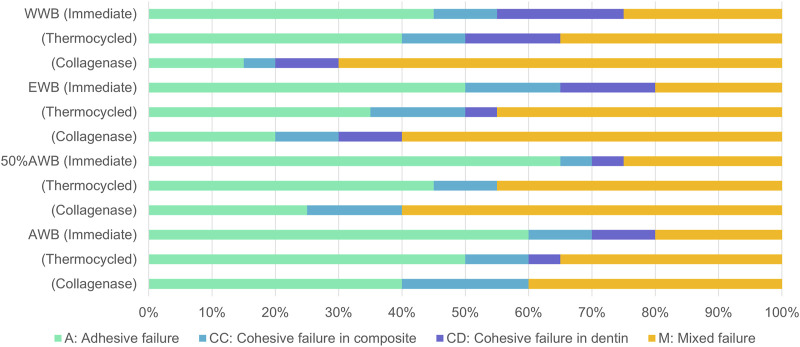
Distribution of failure modes after MTBS test.

**FIGURE 5:**
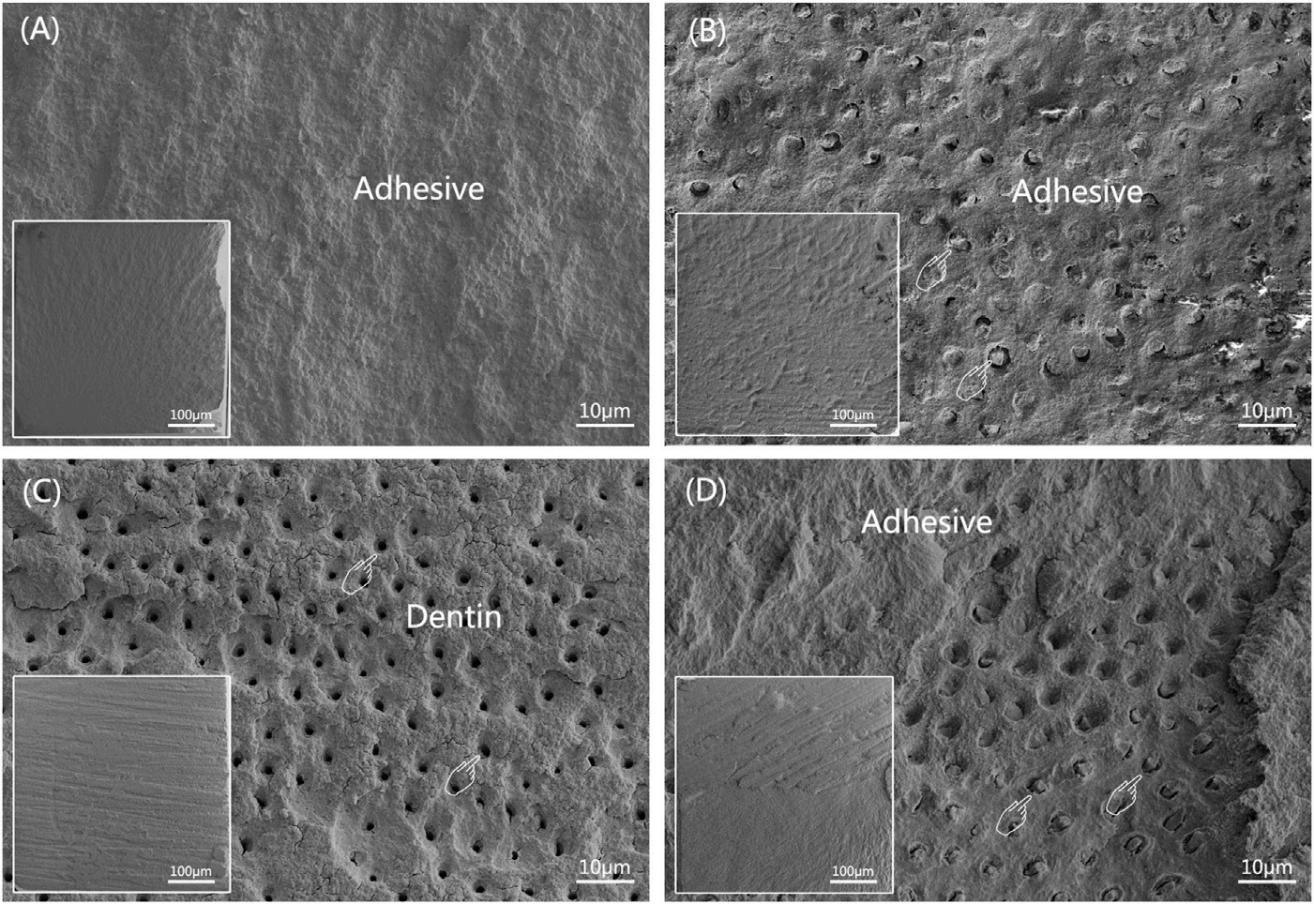
Representative FESEM images of dentin sides of fractured surfaces after MTBS test (The overall morphology is shown at the left lower corner of corresponding image). **(A)** adhesive failure; **(B)** cohesive failure in composite, pointers denoting occluded tubules; **(C)** cohesive failure in dentin, pointers denoting open dentinal tubules; **(D)** mixed failure, pointers denoting occluded tubules.

Figure 4 illustrates the frequency distribution of fracture modes for the four groups. Compared to WWB group, the occurrence rate of adhesive failure increased to varying degrees in the other groups. Adhesive failure was the predominant mode in the immediate groups, whereas the occurrence rate of mixed failure showed an increase in the ageing groups. Figure 5 presents the representative FESEM images of the fractured surfaces.

### 3.3 Interfacial nanoleakage evaluation of adhesive-dentin interface

Table 1 exhibits the quantitative data obtained from nanoleakage evaluation. According to the Kruskal-Wallis test results, specimens pretreated with acetone exhibited significantly lower levels of nanoleakage (*p <* 0.05) compared to the control group, irrespective of the ageing method (i.e., thermocycling or collagenase ageing). Similar nanoleakage level was observed in the WWB group and 50%AWB group (*p >* 0.05).

**TABLE 1:** Percentage distribution of nanoleakage scores from each group for immediate, 10,000 runs of thermocycled and after 1-month collagenase ageing.

Groups	Pretreatment	Score percentage (%)					Statisticaldifference
		0	1	2	3	4	
Immediate	WWB	0	0	55	45	0	ab
	EWB	15	50	30	0	5	d
	50%AWB	0	20	55	25	0	bc
	AWB	10	55	30	5	0	d
Thermocycling	WWB	0	0	35	65	0	ab
	EWB	5	45	50	0	0	d
	50%AWB	0	5	40	55	0	ab
	AWB	5	65	30	0	0	d
Collagenase ageing	WWB	0	0	15	65	20	a
	EWB	5	0	55	30	10	ab
	50%AWB	0	5	25	50	20	a
	AWB	0	50	35	10	5	cd

The Kruskal-Wallis test with Dunnett’s *post hoc* test. Groups with the same letters are not statistically different (*p >* 0.05), *n* = 20.

**FIGURE 6:**
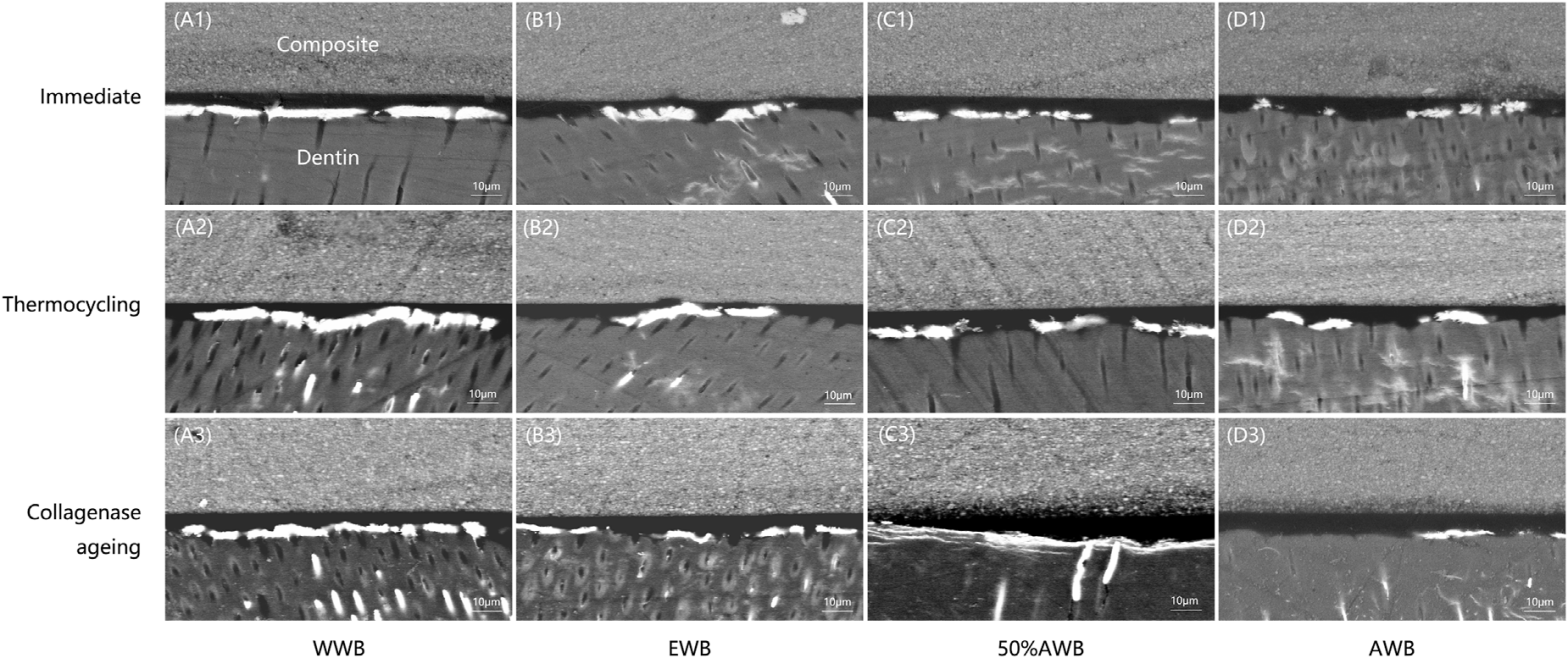
Representative FESEM images of interfacial nanoleakage expression at hybrid layer for each group. Images of **(A1-D1)**, **(A2-D2)**, and **(A3-D3)** represents nanoleakage for immediate, thermocycling, and collagenase ageing evaluation, respectively. Images of **(A1-A3)**, **(B1-B3)**, **(C1-C3)**, and **(D1-D3)** represents the WWB, EWB, 50%AWB, and AWB groups, respectively.

Figure 6 shows representative FESEM micrographs of interfacial nanoleakage. Regardless of the ageing methods (immediate, thermocycling or collagenase ageing), the control and 50%AWB group showed thick and continuous silver precipitates along the adhesive-dentin interface, part of which infiltrated into the dentin tubules. However, sparse and interrupted distribution of silver particles along the interface of dentin and adhesive was observed in the EWB and AWB group. Furthermore, there were no silver granules observed within the dentin tubules, with the AWB group being particularly noteworthy in this regard. The silver uptake changes were not significant after thermocycling (Figure 6A2-D2) or collagenase ageing (Figure 6A3-D3) compared to immediate groups (Figure 6A1-D1).

### 3.4 Zymography of the hybrid layer

**FIGURE 7:**
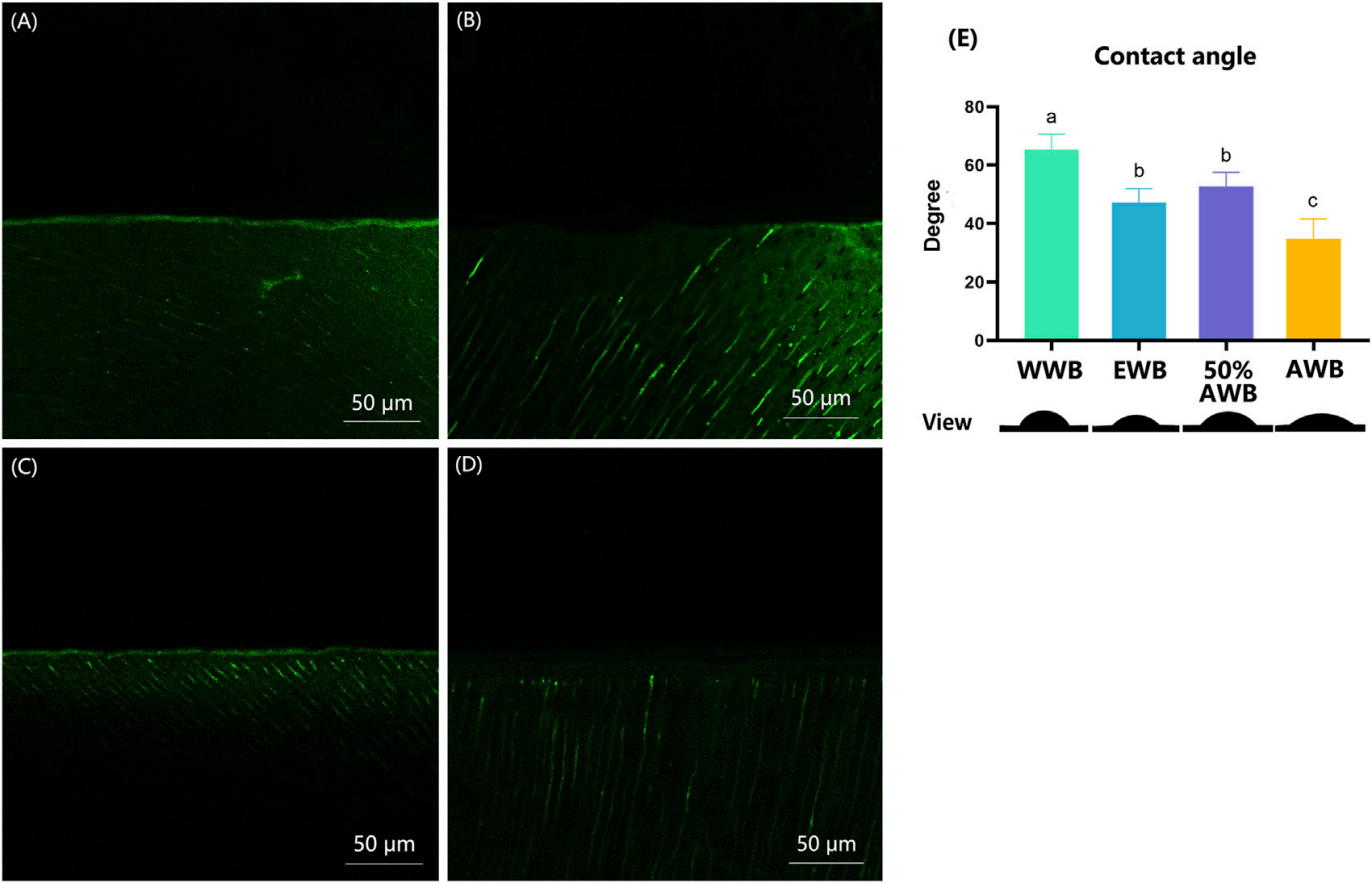
**(A–D)** Characteristic CLSM pictures from *in situ* zymography stained by fluorescent DQ gelatin conjugate. **(A)** water wet-bonding (WWB, **(A)**; **(B)** ethanol wet-bonding group (EWB, **(B)**; **(C)** 50% (v/v) acetone aqueous solution (50%AWB, **(C)**; **(D)** 100% acetone solution (AWB, **(D)**. **(E)** The degree of adhesive contact angle and respective views of the different pretreatment groups on the demineralized dentin surfaces. Groups with different letters are statistically different (*p <* 0.05). Real representative image of each group’s anterior view was displayed below accordingly.

Figures 7A–D shows the typical CLSM images from the control or different pretreatment groups, which indicates the activity of endogenous proteases within the hybrid layers. In the WWB group, there was a substantial presence of green fluorescence within the hybrid layer, signifying extensive hydrolyzation of the DQ gelatine conjugate in this area compared to other groups. Conversely, the 50%AWB group displayed less green fluorescence within the hybrid layer, though the level of fluorescence was higher than that observed in the EWB and AWB groups. The EWB group showed a minimal level of green fluorescence, whereas the AWB group exhibited almost no green fluorescence along the hybrid layer.

### 3.5 Surface contact angle measurements

Figure 7E shows the contact angle values for each group along with their respective views. Real representative images of each group were displayed below the contact angle values. Among the four groups, the WWB group demonstrated the highest contact angle (*p <* 0.05). Both the EWB and 50%AWB groups exhibited significantly lower contact angles compared to the WWB group, yet notably higher angle than the AWB group (*p <* 0.05). The AWB group demonstrated the lowest contact angle (*p <* 0.05), indicating that the acetone pretreatment improved the permeability of the dental adhesive into the dentin surface.

## 4 Discussion

The current study assessed the impact of acetone wet-bonding (AWB) on enhancing the bonding strength of adhesive-dentin interface. The application of acetone and a 50% aqueous solution of acetone on the dentin surface as a pretreatment was found to increase the immediate bond strength. This enhanced bond strength was maintained even after undergoing different ageing methods, such as 10,000 runs of thermocycling and 1 month of collagenase ageing. Furthermore, the use of the AWB method resulted in a reduction in nanoleakage compared to the water wet-bonding technique, regardless of whether the sample underwent thermocycling or collagenase ageing. Additionally, acetone pretreatment suppressed the activity of endogenous proteases, and notably, decreased the contact angle of the etched dentin surface to dental adhesives. Consequently, the null hypotheses were disproved.

During the development of dentin bonding techniques, it was discovered that moisture on the dentin surface plays an essential role in achieving high dentin bond strength (Yiu et al., 2005). Hence, the WWB technique was introduced, which involves the use of acid etching to markedly reduce the water contact angle on the dentin surface (Rosales-Leal et al., 2001). However, the low vapor pressure of water relative to other solvents necessitates a longer air-drying time, and residual water can cause hydrolytic degradation, making it less than ideal (Ekambaram et al., 2015; Jacobsen and Soderholm, 1995; E. L; Pashley, Zhang et al., 1998). As a result, alternative solvents such as ethanol were introduced (D. H. Pashley et al., 2007). Of all the bonding solvent employed in dental adhesives, acetone has the highest vapor pressure and effectively eliminates moisture from the demineralized dentin matrix (Van Landuyt et al., 2007). Additionally, acetone exhibits lower viscosity compared to ethanol, allowing it to better penetrate the demineralized dentin (Ekambaram et al., 2015; Besinis et al., 2016).

Contact angle measurement can be utilized to quantify the extent of liquid spreading on a surface. (Marshall et al., 2010). However, dentin is a non-ideal surface because of its composition and surface roughness (Besinis et al., 2016). In the wet-bonding technique process, the dentin was acid etched, water rinsed and blot-dried before the surface is treated with solvents and dental adhesives (D. H. Pashley et al., 2007). Thus, the real wettability of dentin surface after treatment is actually measurement of contact angle of the dentin surface to dental adhesives. In order to correctly measure the dentin surface wettability, a thin solvent film, according to different pretreatment groups, was cautiously left on the dentin surfaces to mask surface irregularities like smear layer and dentinal tubules, resulting in a smoother surface. If the dentin surface was dry, then the irregularities would impact the contact angle measurements necessitating the need for correction by applying the Wenzel equation (Wenzel, 1936). In this study, this method was undertaken to improve the accuracy of contact angle measurement.

In the acetone wet-bonding (AWB) technique, dentin is acid-etched and then rinsed with water, after which acetone solution is applied to interact with the residual water in the demineralized dentin. This process results in the displacement of water and transportation of adhesive monomers into the dentin collagen matrix with the help of acetone. Consequently, it enhances the dentin surface’s wetting properties and improves the infiltration of adhesive (D. H. Pashley and Carvalho, 1997). In the present study, the application of acetone on the dentin led to a significant decrease in contact angle, indicating improved wettability. While pretreatment with ethanol and acetone aqueous solution also reduced the contact angle compared to water, the effect was not as pronounced as that of pure acetone. This “activation effect” of the dentin surface by the AWB technique helps to lower the surface tension of the dentin collagen matrix, creating better compatibility with primer and adhesive resins. This, in turn, facilitates the improved infiltration of adhesive monomers into the etched dentin (Besinis et al., 2016; D. H; Pashley and Carvalho, 1997). Notably, the contact angle was lowest in the AWB group, suggesting that acetone is the most effective solution for enhancing dentin wettability compared to other solvents.

The infiltration of monomer into the collagen matrix after etch-and-rinse has always been a problem (J. Guo et al., 2017). Deep penetration of the adhesive monomer can enhance the bond strength, as well as reducing the amounts of unprotected collagens which are vulnerable to hydrolysis. The breakdown of demineralized dentin matrices is a procedure that involves the swift and spontaneous establishment of fresh hydrogen bond (H-bonds) among collagen peptides (D. H. Pashley et al., 2007). Hoy’s solubility parameter theory is widely used to evaluate capacity of chemicals to H-bonds (Barton, 1991). D. H. Pashley *et al* calculated Hoy’s solubility parameters of collagen and found that 100% collagen has a Hoy’s δ_h_ value of 14.8 (J/cm^3^)^1/2^ (Agee et al., 2006). Only solvents like water with a δ_h_ value of 40.4 (J/cm^3^)^1/2^ which is higher than collagen have the capacity to disrupt the interpeptide hydrogen bonds, permitting the expansion of the collagen matrix. However, water is volatile and cannot polymerize and most adhesive monomers have δ_h_ value lower than 14.8 which means, in neat form, they cannot expand dry dentin collagen matrix (D. H. Pashley et al., 2007). In order to replace water and transport hydrophobic monomers into the gaps of collagen, amphiphilic solvents are needed. Acetone, which is amphiphilic and possesses qualities like strong permeability, can replace water and make it evaporate quickly, then transport hydrophobic monomers like MDP and hydrophilic monomers like HEMA into the gaps between collagens (Besinis et al., 2016). The immediate bond strength of 50% acetone pretreatment group (50%AWB) is notably higher than that of WWB group (*p <* 0.05), since the acetone aqueous solution can expand the collagen matrix efficiently and help the adhesive monomers infiltrate deeper simultaneously. Also, better penetration of monomers and more thorough replacement of water with the help of acetone lead to the decrease of unprotected collagens and the inhibition of enzyme hydrolysis, resulting in good bond durability. Thus, the bond strength of AWB group exhibited a notable increase compared to the WWB group following thermocycling or collagenase ageing (*p <* 0.05). Furthermore, neither thermocycling nor collagenase ageing led to a substantial decline in bond strength for the 50%AWB and AWB group.

The expression of nanoleakage is commonly utilized as a significant indicator to evaluate the durability and stability of dental bonding due to the fact that the intrusion of bacteria and the penetration of water into voids or channels within the adhesive-dentin interface can result in the deterioration of the hybrid layer (Deng et al., 2014). Kruskal-Wallis test (Table1) indicated that nanoleakage of AWB group significantly decreased in comparison to other groups (*p <* 0.05), irrespective of thermocycling or collagenase ageing. This was further demonstrated by details in Figure 6 which exhibited fewer silver deposition in AWB group. Dentin bonding is a procedure that encompasses the demineralization of dentin surface and refilling of the collagen matrix by adhesive monomers. After infiltration, adhesive monomers help form the mechanical interlock between dentin collagen and the adhesive (Pashley et al., 2003). After demineralization, it is essential to ensure that water and solvents are thoroughly eliminated from the dentin surface before curing to avoid jeopardization of the polymerization of adhesives (Yiu et al., 2005). However, the water is difficult to be removed due to its low vapor pressure (Ekambaram et al., 2015). Other solvents are then needed to replace water and help its evaporation. Acetone, which is called “water chaser”, would be the most efficient solvent because of its vapor pressure as high as 184.8 mmHg at 20 °C (Ekambaram et al., 2015). It would not only help water to evaporate but also facilitate the penetration of adhesive monomers. Therefore, the silver deposition in AWB group, which represents the holes in hybrid layer caused by the residual water after bonding, decreased significantly compared to other groups as shown in Figure 6. R. L. Sakaguchi and J. M. Powers (Sakaguchi and Powers, 2012) insisted on applying multiple coats of acetone-based adhesive due to the potential risk of evaporation during storage. However, as pretreatment solution, this would not be a problem since the concentration of pure acetone solution would not change, although the volume loss during storage is still inevitable.

The deterioration of the dentin collagen matrix can be initiated by internal enzymes such as MMPs and cysteine cathepsins and external enzymes like bacterial collagenase (Mazzoni et al., 2015). Thus, it is necessary to evaluate the activities of exogenous and endogenous enzymes. In this study, *in situ* zymography test of the hybrid layer developed by Breschi was chosen to detect the location of enzyme activities (Mazzoni et al., 2014). This method can reveal the gelatinolytic activity within the hybrid layer, which aligns with areas where collagen is left unprotected owing to incomplete resin infiltration. Results revealed a significant presence of fluorescent green within the control group, affirming the heightened activity of proteases. Conversely, the intensity of fluorescent green diminished progressively when higher concentrations of acetone were applied, suggesting the inhibition of the gelatinolytic activity. The details in Figure 7A disclosed that pretreatment with ethanol and acetone can significantly suppress the activity of endogenous proteases. There was almost none fluorescent green detected in AWB group as shown in Figure 7D. Although no direct evidence shows that acetone could inhibit dentin proteases, this phenomenon may be contributed to the better penetration of adhesive monomers with the help of acetone. After acid etching of dentin surfaces, the collagen left unprotected would be degraded by enzymes, resulting in poor dentin bonding durability (Liu et al., 2011). Since the acetone helps water evaporate, the adhesive monomers were transported deeply into the gaps of the dentin collagen. Subsequently, the collagens were enveloped and protected by adhesive monomers, thereby preventing enzymatic degradation of the collagen. The negative correlation of gelatinolytic activity and bond durability corroborated that enzyme activity could be a contributing factor to the decline in long-term bond strength.

In this study, the implementation of the acetone wet-bonding technique represents a promising approach for reducing contact angle, improving resin monomer penetration and enhancing dentin bond durability. However, limitations cannot be neglected. The high vapor pressure of acetone is a mixed blessing. Although it helps the removal of water from dentin surfaces and improves the infiltration of resin monomers into collagen matrix, the risk of evaporation can impact its longevity on the shelf. Further investigations are warranted to delve in to the underlying mechanisms of how acetone interfere with collagen hydrolytic degradation.

## 5 Conclusion

This study posits that the acetone wet-bonding (AWB) technique exhibits the capacity to sustain bond strength over an extended duration. Additionally, it demonstrates efficacy in improving dentin wettability to dental adhesives, facilitating adhesive monomer penetration, mitigating collagen exposure, decreasing nanoleakage, and attenuating collagen degradation. Within the confines of this *in vitro* investigation, acetone wet-bonding emerges as a promising strategy for prolonging the lifespan of adhesive restorations.

## Data Availability

The original contributions presented in the study are included in the article/Supplementary material, further inquiries can be directed to the corresponding authors.
